# The engineered expression of secreted HSPB5-Fc in CHO cells exhibits cytoprotection in vitro

**DOI:** 10.1186/s12896-021-00700-y

**Published:** 2021-06-14

**Authors:** Jing Li, Jingjing Yu, Wenxian Xue, Huili Huang, Longjun Yan, Fan Sang, Shuangshuang An, Jing Zhang, Mingli Wang, Jun Zhang, Hui Li, Xiukun Cui, Jiang He, Yanzhong Hu

**Affiliations:** 1 Joint National Laboratory for Antibody Drug Engineering, The First Affiliated Hospital of Henan University, School of Basic Medical Sciences, Henan University, Jin-Ming Road, Kaifeng, 475004 China; 2 Kaifeng Key Lab for Cataract and Myopia, Institute of Eye Disease, Kaifeng Central Hospital, Kaifeng, China; 3 Center for Molecular Medicine, Xiangya Hospital, Central South University, Changsha, China; 4 Department of Ophthalmology, First Affiliated Hospital of Zhengzhou University, Zhengzhou, China

**Keywords:** HSPB5, Affinity purification, Chaperone activity, polyQ, Apoptosis

## Abstract

**Background:**

HSPB5 is an ATP-independent molecular chaperone that is induced by heat shock or other proteotoxic stresses. HSPB5 is cytoprotective against stress both intracellularly and extracellularly. It acts as a potential therapeutic candidate in ischemia-reperfusion and neurodegenerative diseases.

**Results:**

In this paper, we constructed a recombinant plasmid that expresses and extracellularly secrets a HSPB5-Fc fusion protein (sHSPB5-Fc) at 0.42 μg/ml in CHO-K1 cells. This sHSPB5-Fc protein contains a Fc-tag at the C-terminal extension of HSPB5, facilitating protein-affinity purification. Our study shows that sHSPB5-Fc inhibits heat-induced aggregation of citrate synthase in a time and dose dependent manner in vitro. Administration of sHSPB5-Fc protects lens epithelial cells against cisplatin- or UVB-induced cell apoptosis. It also decreases GFP-Htt^ex1^-Q74 insolubility, and reduces the size and cytotoxicity of GFP-Htt^ex1^-Q74 aggregates in PC-12 cells.

**Conclusion:**

This recombinant sHSPB5-Fc exhibits chaperone activity to protect cells against proteotoxicity.

**Supplementary Information:**

The online version contains supplementary material available at 10.1186/s12896-021-00700-y.

## Background

HSPB5 is a member of a family of small heat shock proteins, a class of ATP-independent molecular chaperones with a conserved alpha-crystallin domain that protects cellular proteostasis by delaying the formation of insoluble protein aggregates through their interactions with destabilized, aggregate-prone proteins [1–4]. Acting as a chaperone, HSPB5 regulates many cellular processes, such as skeletal protein reorganization [2], heat shock response [5], exosome secretion [6], protein metabolism [7], anti-apoptosis [5] and anti-senescence. Dysfunction of HSPB5 is closely associated with proteinopathies (such as Alzheimer’s disease, cataracts, muscle degeneration, heart atrophy, diabetes), tumorigenesis and tumor metastasis [8–11] and autoimmune diseases [12]. Therefore, HSPB5 is considered a valuable therapeutic target.

HSPB5 protein consists of 175 amino acids, and is constitutively expressed in most tissues at physiological conditions [13]. Its expression is increased by heat shock or other proteotoxic stresses. The amino acids of HSPB5 functionally constitute three regions, a N-terminal region (1–66), a central conserved alpha-crystallin domain (aa 66–149, ACD) and a C-terminal region (149–175) [14]. The C-terminal region contains a conserved IXI motif (aa159–161) and a flexible C-terminal extension [15]. HSPB5 exerts chaperone activity as monomers and dimers rather than as oligomers [16]. HSPB5 inhibits both amorphous and fibrillar protein aggregation through physical interaction [17], and its chaperone activity is regulated by its intramolecular interaction between the ACD domain and the IXI motif [18, 19], phosphorylation [20], temperature [21], pH [22], and oligomerization with other small heat shock proteins (e.g., HSPB4, HSPB1 and HSPB6) [23]. About 115 proteins are reported to interact with or regulated by HSPB5.

HSPB5 exerts its cytoprotective roles both intracellularly and extracellularly. HSPB5 is secreted extracellularly via a vesicle-mediated unconventional protein secretion system that is regulated by TEMD10 and stress [24]. HSPB5 is secreted by retinal pigment epithelial cells and its secretion is regulated by the phosphorylation of serine 59 in HSPB5 [25, 26]. Administration of recombinant HSPB5 proteins attenuates retinal injury induced by oxidative stress [27, 28], protects heart from ischemic injury [29] and delays cataract formation in animal models [30]. The recombinant HSPB5 used in research is from heterogenous expression in the engineered *E. coli* BL21 [31, 32]. Although this bacteria-heteroexpressed HSPB5 exerts chaperone activity, it cannot completely imitate the HSPB5 secreted by mammalian cells due to a lack of posttranslational modification [33, 34]. Earlier, it has been reported that human recombinant lactoferrin, carrying a humanized glycosylation, displays selective antiproliferative effects on cancer cells [35]. This research suggests that natural recombinant proteins possess beneficial biological activities on human health.

In this paper, we generated a recombinant construct that can express and secret a sHSPB5-Fc fusion protein to the supernatant when this construct is transfected into CHO-K1 cells. This sHSPB5-Fc fusion protein, which exists predominantly as dimer and trimer and is regulated by N-linked glycosylation, inhibits Citrate synthase (CS) protein aggregation under heat shock in vitro. Administration of sHSPB5-Fc protein reduces the aggregate size and the cytotoxicity of GFP-Htt^ex1^-Q74 in PC-12 cells, and protects lens epithelial cells against cisplatin- and UVB-induced cell apoptosis. These results suggest that this sHSPB5-Fc fusion protein exhibits both chaperone and cytoprotective roles in vitro, and is a possible candidate for studying of its therapeutic potentials.

## Results

### The expression and purification of recombinant HSPB5-Fc proteins

**Fig. 1:**
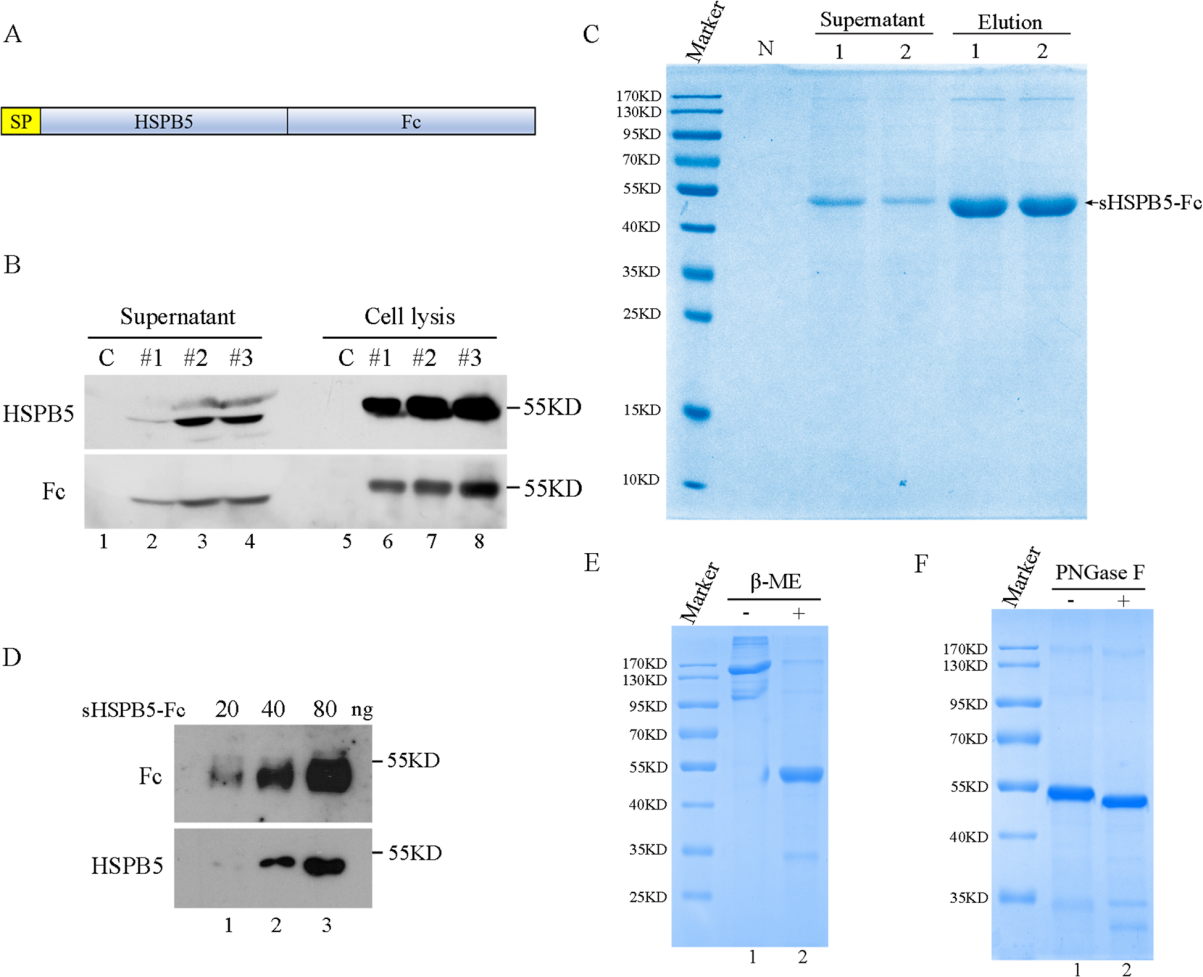
The expression of recombinant sHSPB5-Fc protein. **A**, Schematic of the sHSPB5-Fc construct. SP: signal peptide. Fc: Fc-tag. **B**, Immunoblot of the expression of HSPB5-Fc in the supernatant and cell lysates with antibodies against the Fc-tag and HSPB5. Lane 1: CHO-K1 cells with empty vector; lanes 2–4: CHO-K1 cells expressing three psHSPB5-Fc constructs (#1, #2, #3) respectively. **C**, Coomassie blue stain of sHSPB5-Fc protein purified from CHO-K1 supernatant (psHSPB5-Fc #2 construct). Supernatants: 1 and 2 represented non purified sHSBP5-Fc in supernatants. Elution 1 and 2: represented purified sHSPB5-Fc. **D**, Immunoblot of the purified sHSPB5-Fc in C with antibodies against Fc-tag and HSPB5. Lanes 1–3 represents 20, 40 and 80 ng sHSPB5-Fc proteins respectively. **E**, Coomassie blue staining sHSPB5-Fc protein that was treated with SDS-loading buffer with (lane 2) or without β-mercaptoethanol. **F**. Coomassie blue staining sHSPB5-Fc protein that was treated with PNGase F

To express a secreted HSPB5 (sHSPB5) in mammalian cells, the constructs of psHSPB5-Fc (Fig. 1A) and empty vector were transiently transfected into CHO-K1 cells individually. The supernatants and cell lysates were immunoblotted with antibodies against Fc-tag or HSPB5 respectively. Anti-Fc and anti-HSPB5 antibodies detected sHSPB5-Fc in both supernatants and cell lysates (Fig. 1B). The protein size of sHSPB5-Fc in the supernatant was smaller than that in the cell lysate, suggesting that the signal peptide of sHSPB5-Fc was cleaved. CHO-K1 is a common cell line used to express recombinant proteins in the medical industry. We subcloned a CHO-K1-sHSPB5-Fc cell line that could produce 0.42 mg/ml of sHSPB5-Fc in the supernatant. The sHSPB5-Fc protein was purified from the supernatants with protein-A affinity column, and the quality of purified sHSPB5-Fc protein was verified with Coomassie blue stain and western blot (Fig. 1C-D), which showed a protein size of 53 K Dalton, consistent with the predicted size. HSPB5 contains a crystalline domain by which HSPB5 forms homo-dimer, trimer and polyoligomers in cells [16]. Fc-tag also forms dimers through the disulfide bond. To characterize the status of sHSPB5-Fc, the sHSP5-Fc protein was heated in protein loading buffer with or without β-Mercaptoethanol (β-ME). The Coomassie blue staining results showed that majority of sHSPB5-Fc protein existed in dimers and trimers, but less sHSP5-Fc forms oligomers in the native loading buffer. The reducing agent β-ME minimized the size of sHSPB5-Fc from oligomers to monomer (Fig. 1E). These results indicated that sHSPB5-Fc protein exists predominantly in dimer and trimer. In addition, we studied that whether sHSPB5-Fc is regulated by glycosylation by treating the sHSPB5-Fc protein with PNGase F, an enzyme catalyzing the cleavage of N-linked polysaccharides from glycoproteins. The results showed that PNGase F increased the mobility of sHSPB5-Fc in a SDS-PAGE gel, suggesting that sHSPB5-Fc undergoes glycosylation (Fig. 1F).

### Recombinant sHSPB5-Fc experts chaperone activity in vitro

**Fig. 2:**
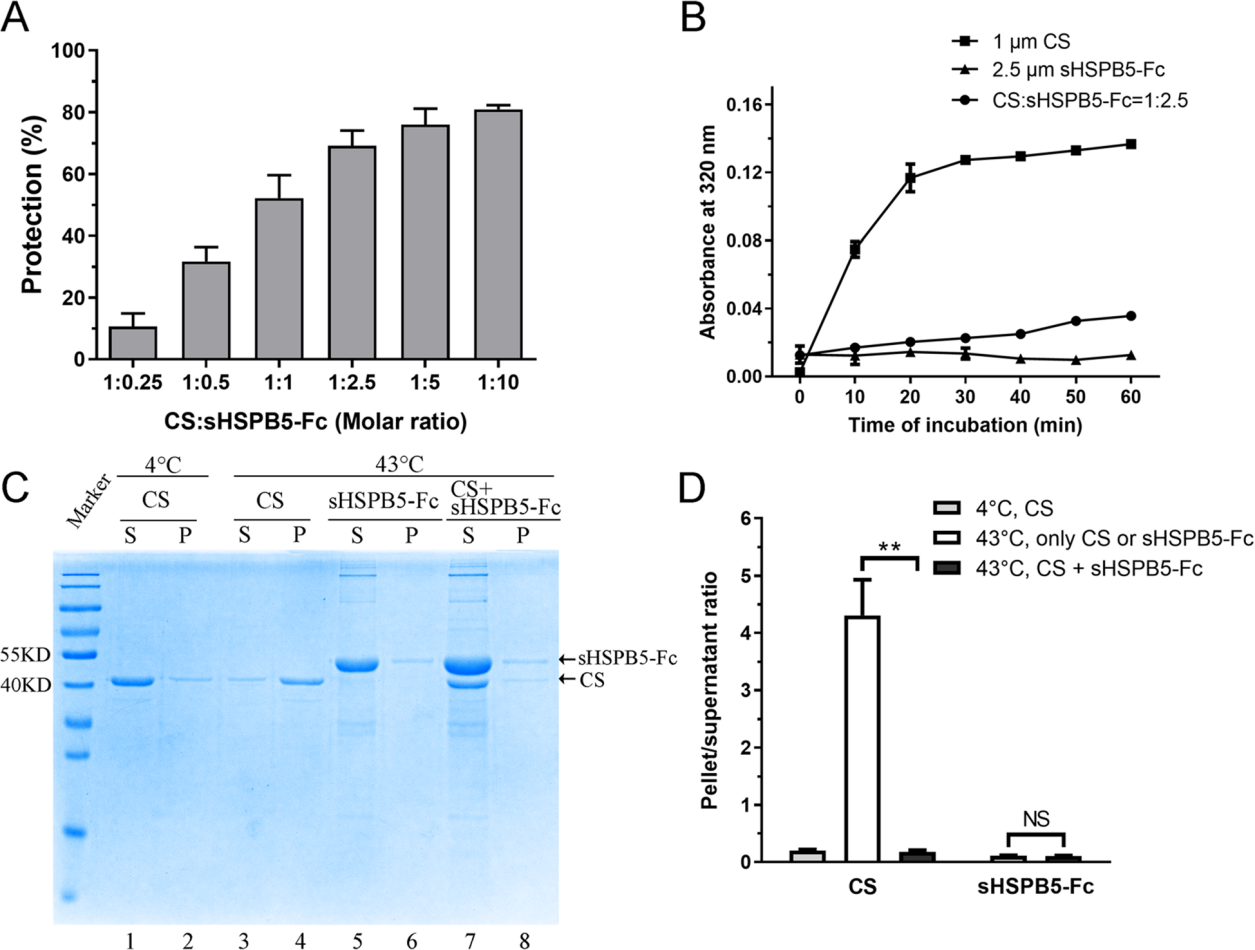
The recombinant sHSPB5-Fc exhibits chaperone activity in vitro. **A**, sHSPB5-Fc inhibits heat-induced aggregation of CS in a dose-dependent manner. CS alone or combined with sHSPB5-Fc at a series of molar ratios were incubated at 43 °C in pH 7.4, 40 mM HEPES for 1 h. The absorbance at 320 nm was measured using a microplate reader of multi-wavelength measurement system (Thermo Fisher Scientific). The protection (%) = (1-A320_CS + sHSPB5-Fc_/A320_CS_) × 100%. **B**, Time course to assess the chaperone activity of sHSPB5-Fc to substrate CS. **C**, Chaperone activity of sHSPB5-Fc on heat-induced aggregation of CS protein using the soluble and insoluble fraction assay. The soluble fraction was separated from the insoluble fraction by centrifugation. The soluble and insoluble proteins were detected with Coomassie blue staining. S, soluble protein. P, pellet of aggregated protein. **D**, Quantification of pellet versus soluble protein ratio in (**C**). The densitometry of CS and HSPB5-Fc proteins in the soluble fraction and pellet were measured using Image J software. The gray value ratios of pellet versus soluble protein are shown in the bar graph. Column 1: the ratio of CS alone at 4 °C. Column 2: the ratio of CS alone at 43 °C. Column 3: the ratio of CS when combined with HSPB5-Fc at 43 °C. Column 4: the ratio of HSPB5-Fc alone at 43 °C. Column 5: the ratio of HSPB5-Fc when combined with CS at 43 °C

HSPB5 exerts chaperone activity by interacting with and inhibiting protein aggregates in an ATP-independent manner [13]. Citrate synthase (CS) proteins can form protein aggregates under heat shock condition, and this feature has been used to measure the chaperone activity of small heats shock proteins (e.g., HSPB5 or HSPB1) in vitro [13]. We determined the chaperone activity of sHSPB5-Fc by using heat-induced aggregation of CS in vitro. We incubated CS protein alone or together with sHSPB5-Fc at the indicated ratios (Fig. 2A) at 43 °C in 40 mM HEPES buffer (pH 7.4) for 1 h. The aggregated proteins were measured with spectrometry at OD 320 nm. The results showed that sHSPB5-Fc increased CS protein solubility under heat shock conditions in a dose-dependent manner (Fig. 2A). Using the ratio of CS:sHSPB5-Fc of 1:2.5, we further studied the time course influence on sHSPB5-Fc chaperone activity. The results showed that CS protein alone rapidly formed aggregates at 43 °C within 20 min, after which prolonged heat shock time did not increase CS protein aggregation. This suggested that aggregation plateaued after 20 min. sHSPB5-Fc significantly inhibited aggregation of CS protein at 43 °C. sHSPB5-Fc protein alone did not form aggregates under this heat shock condition (Fig. 2B). Furthermore, the chaperone activity of sHSPB5-Fc was tested in both the soluble and insoluble fractions. The solutions that contain CS protein or sHSPB5-Fc alone, or a mixture of CS and sHSPB5-Fc at a ratio of 1:2.5, were treated at 43 °C for 1 h followed by centrifugation at 15000 g for 15 min to separate the pellets from supernatants. The proteins in the pelleted and soluble fractions were analyzed on SDS-PAGE gel and stained with Coomassie blue (Fig. 2C). The densitometry of protein bands was quantitated using image J software and the ratio of pelleted protein vs soluble proteins was quantified (Fig. 2D). The results showed that most of the CS proteins were in the soluble fraction compared to that in the pelleted fraction at 4 °C, and the ratio of pelleted vs soluble CS protein is 0.27 (Fig. 2C, lanes 1 and 2). However, at 43 °C, the bulk of CS proteins was detected in the pelleted fraction (Fig. 2C, lanes 3 and 4) with a ratio of 4. 2 (Fig. 1D). sHSPB5-Fc reduced the amount of CS proteins in the insoluble fraction (Fig. 2C, lanes 7 and 8), with a ratio of 0.34 (Fig. 2D). sHSPB5-Fc always remained in the soluble fraction under heat shock regardless of the presence of CS protein (Fig. 2C, lanes 5, 6, 7 and 8). Taken together, these results suggest that the recombinant sHSPB5-Fc protein can exhibit chaperone activity in vitro.

### The recombinant sHSPB5-Fc protein protects cells from stress-induced apoptosis in vitro

**Fig. 3:**
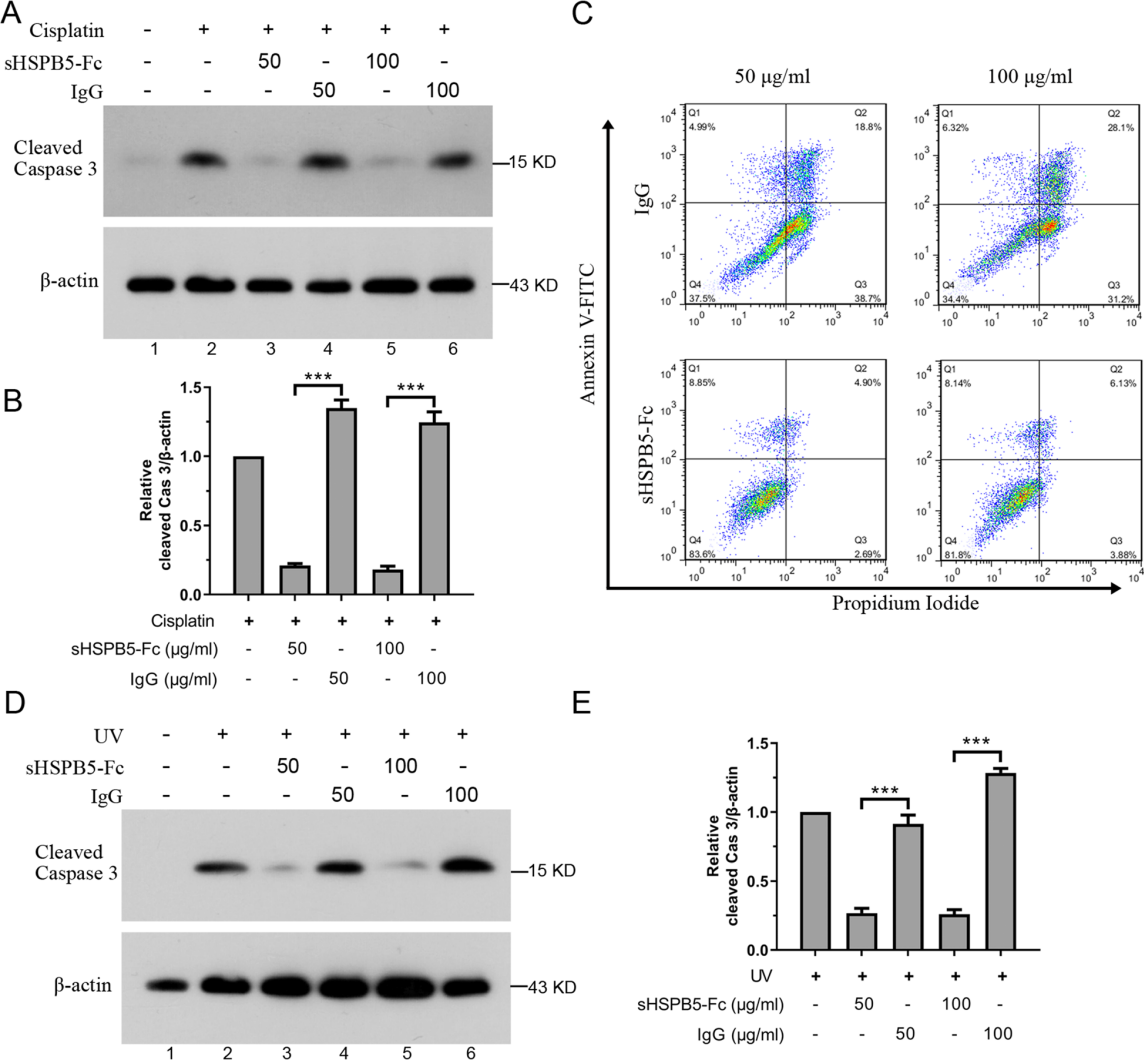
Recombinant sHSPB5-Fc exhibits anti-apoptosis activity. **A**, mLEC cells were incubated with 10 μM cisplatin alone or 10 μM cisplatin together with 50 and 100 μg/ml sHSPB5-Fc or human IgG (used as a control) for 24 h. The cleaved Caspase 3 was immunoblotted. **B**, Quantification of cleaved Caspase 3 in A with Image J. The quantification of cleaved Caspase 3 versus β-actin was accounted for the relative levels of Caspase 3 activity. **C**, The cells were treated in same way as in A, the cells were stained with FITC-Annexin-V and PI followed by analysis in flow cytometry. The apoptosis percent was shown in Q2 and Q3. The percentage of Q3 means the early apoptotic cells and that of Q2 means the late apoptotic cells. **D**, sHSPB-5-Fc attenuates UVB-induced cell apoptosis in vitro. mLEC cells were treated with 3 J/m^3^ UVB (lanes 2–6) followed by recovery in complete media alone (lane 2) or in media containing 50 or 100 μg/ml sHSBP5-Fc (lanes 3 and 5) or human IgG as negative control (lanes 4 and 6) for 24 h. The activated caspase 3 was immunoblotted. **E**, Quantification of cleaved Caspase 3 in (**D**) with Image J. The quantification of cleaved Caspase 3 versus β-actin was accounted for the relative levels of Caspase 3 activity. The data were from three independent experiments. Student’s t-test was used for statistical analysis. **p < 0.01, ***p < 0.0001

Administration of recombinant HSPB5 was reported to protect RPE cells against stress-induced apoptosis [28]. To test the anti-apoptotic activity of sHSPB5-Fc, the mouse lens epithelial cell line (mLEC) was cultured in media containing 50 or 100 μg/ml of sHSPB5-Fc followed by treatment with sham (DMSO) or 10 μM cisplatin for 24 h. Human IgG was used as control. The cisplatin is a common chemotherapy agent that induces both tumor and normal cells’ apoptosis. Apoptosis was measured by using flow cytometry to identify cells that stained positive for Annexin V/PI and immunoblotting those cells for active caspase 3. The immunoblots showed that sHSPB5-Fc can significantly inhibit cisplatin-induced caspase 3 activation at both the 50 and 100 μg/ml concentrations (Fig. 3A, lanes 3 and 5, and Fig. 3B). Administration of normal immunoglobulin, which is used as a negative control, did not impact cisplatin-induced caspase 3 activation. The flow cytometry results showed that cisplatin itself increased the percentage of cells with double annexin-V/PI positive, but this effect was inhibited by sHSPB5-Fc (Fig. 3C). In addition, we tested the protective role of sHSPB5-Fc protein on UVB-induced mLEC cell apoptosis. The mLEC was treated with 3 J/m^3^ UVB followed by recovery in media containing sham (IgG) or 50 and 100 μM sHSPB5-Fc for 24 h. The activated caspase 3 was tested with immunoblotted. The results showed that sHSPB5-Fc attenuated UVB-induced caspase 3 activation (Fig. 3D-E). Taken together, our data suggest that the recombinant sHSPB5-Fc can exhibit anti-apoptotic activity.

### HSPB5-Fc can decrease the aggregation and cytotoxicity of GFP-Htt^ex1^-polyQ74 in PC-12 cells

**Fig. 4:**
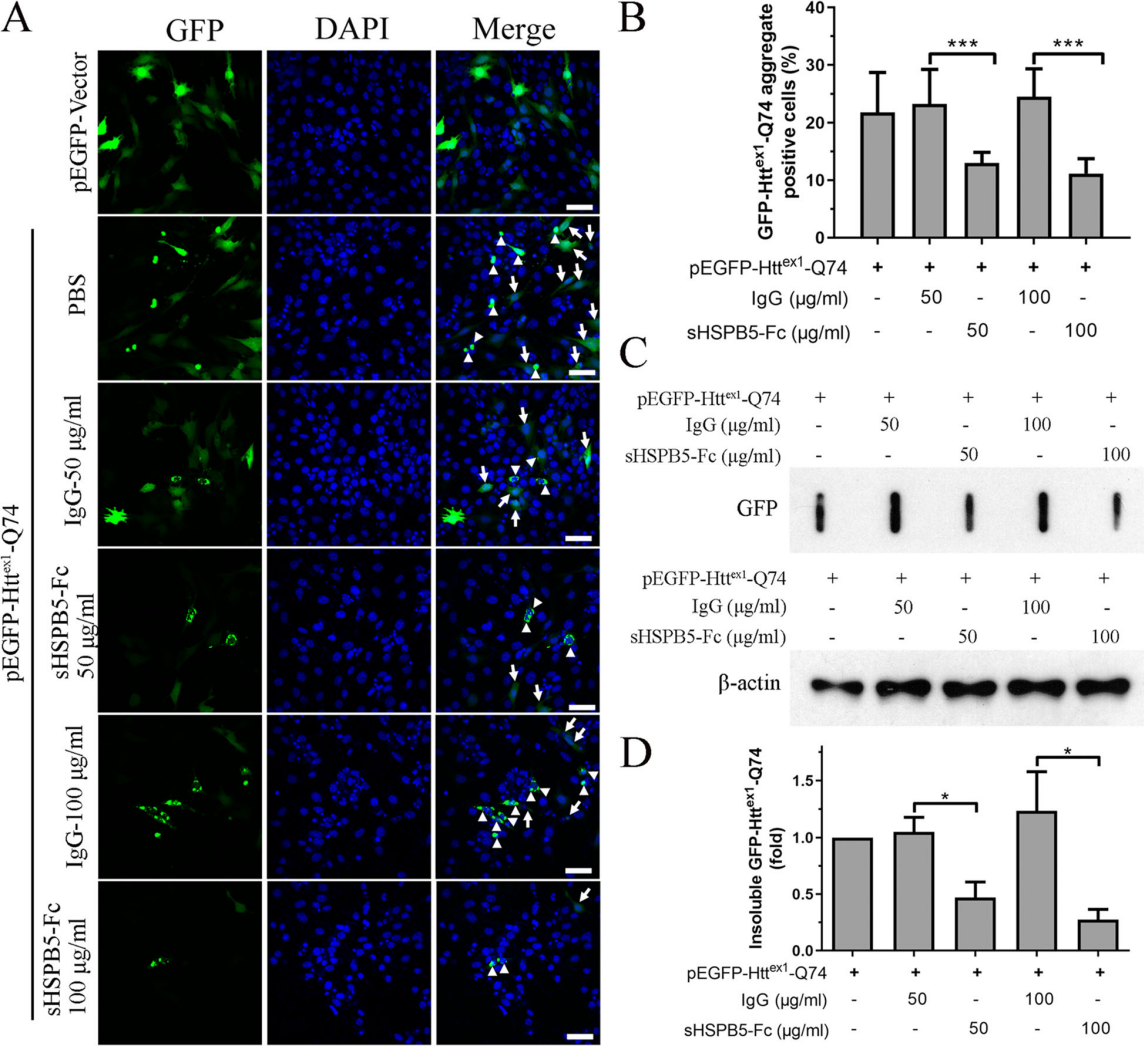
sHSPB5-Fc decreases the aggregation of GFP-Htt^ex1^-Q74 in PC-12 cells. **A**, Fluorescent image of PC-12 cells that express GFP alone or GFP-Htt^ex1^-Q74. The cells were treated with Human IgG (control) or with 50 or 100 μg/ml of sHSPB5-Fc for 24 h. The arrow indicates soluble GFP-Htt^ex1^-Q74. The arrowhead indicates insoluble GFP-Htt^ex1^-Q74. Scale bars are 50 μm. **B**, The percentage of cells with GFP-Htt^ex1^-Q74 aggregates in **A**. Three hundred GFP positive cells of each analysis were analyzed. **C**, Determination of the anti-aggregation properties of sHSPB5-Fc using the membrane-filtration assay. Cell lysates that were treated in same way as in **A** were immunoblotted using a 0.2 μm cellulose acetate (CA) membrane on a slot bot apparatus. The insoluble GFP-Htt^ex1^-Q74 protein on the membrane was immunoblotted with GFP antibody. **D**, The quantification of protein bands in C versus β-actin were measured with Image J software. Student’s t-test was used for statistical analysis. *p < 0.05, **p < 0.01, ***p < 0.0001

Huntington’s disease is a type of neurodegenerative disease caused by the aggregation of huntingtin proteins containing multiple polyQ sequence repeats. Direct inhibition of polyQ aggregation by lefunomide or teriflunomide or by indirectly activating HSF1-mediated heat shock response improves PolyQ-induced neuropathy [36, 37]. It was reported that increased HSPB5 expression in mice improves Huntington disease by reducing poly-Q mediated mHtt aggregation [38]. Thus, we further tested this cytoprotective effect of sHSPB5-Fc in vitro. PC-12 cell line, a neuroblastoma cell line, was transiently transfected with plasmid pEGFP-Htt^ex1^-Q74. The cells were then incubated with media containing sHSPB5-Fc at concentrations of 50 and 100 μg/ml or control IgG for 24 h. The results showed that the cells containing pEGFP vector alone expressed GFP protein evenly in both the cytosol and nucleus (Fig. 4A). In contrast, the cells that were transfected with pEGFP-Htt^ex1^-Q74 formed GFP-Htt^ex1^-Q74 aggregates (Fig. 4A). The administration of sHSPB5-Fc proteins significantly reduced the number of cells with GFP-Htt^ex1^-Q74 aggregates (Fig. 4A-B). To determine whether sHSPB5-Fc decreases the insolubility of GFP-Htt^ex1^-Q74 in PC-12 cells, a membrane-filtration assay was performed. The cells were treated in the same way as that in Fig. 4A, and then lysed in NP-40 lysis buffer. The pellets were treated with SDS buffer. The SDS-treated NP40-insoluble proteins were pass through a 0.2 μm cellulose acetate membrane using a slot blot apparatus followed by immunoblotting with anti-GFP antibody. The results showed that sHSPB5-Fc protein can significantly reduce the amount of GFP-Htt^ex1^-Q74 left on the cellulose acetate membrane (Fig. 4C and D), implying that sHSPB5-Fc can decrease GFP-Htt^ex1^-Q74 aggregation.

**Fig. 5:**
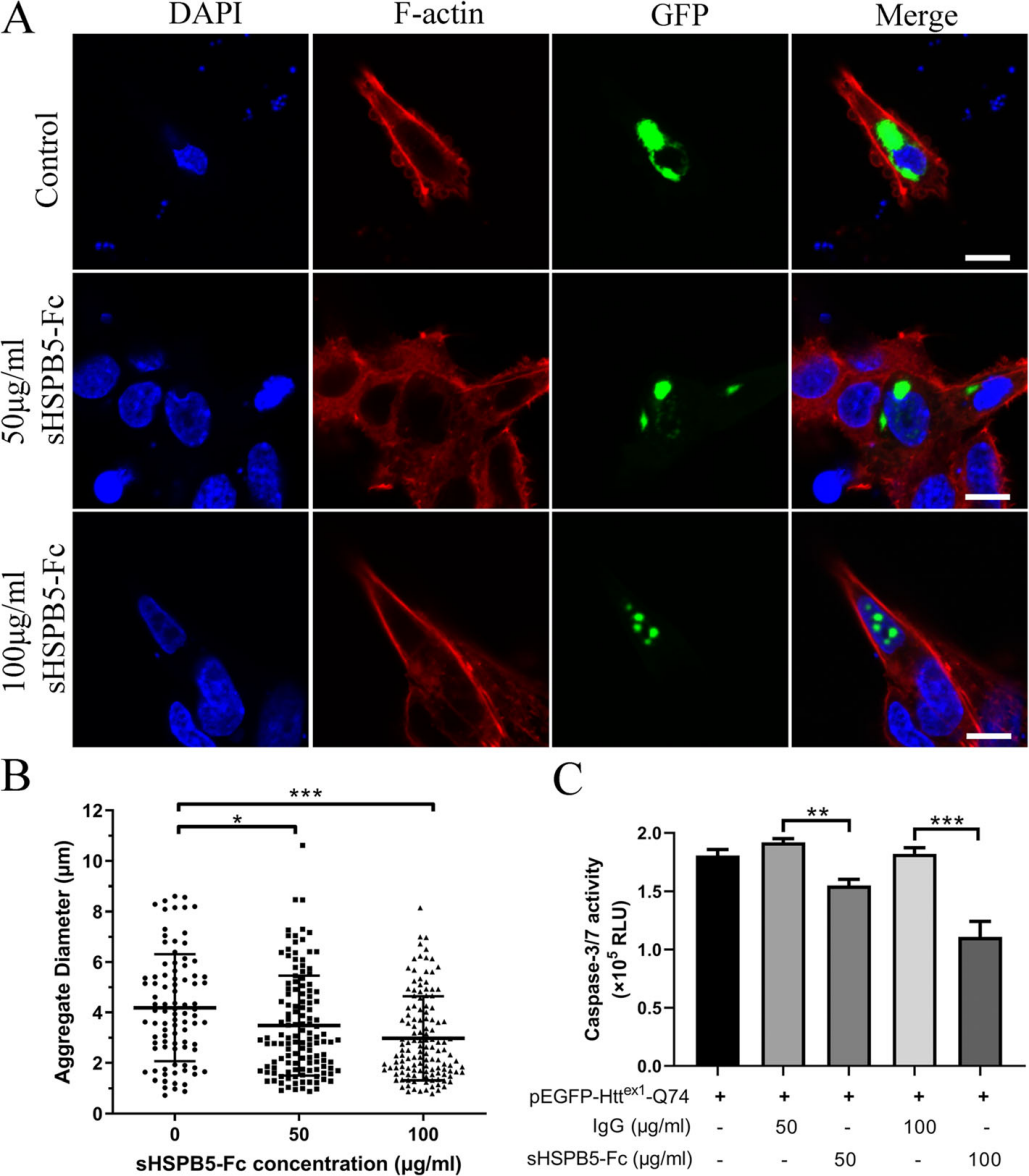
sHSPB5-Fc reduces the size and cytotoxicity of GFP-Htt^ex1^-Q74 aggregates in PC-12 cells. **A**, PC-12 cells that express GFP-Htt^ex1^-Q74 were treated with IgG control or sHSPB5-Fc. The cells were fixed with 4% PFA and stained with phalloidin (for F-actin) and DAPI (Nuclei). The diameters of insoluble GFP-Htt^ex1^-Q74 aggregates were measured with a confocal microscope software. Fifty different views were photographed for aggregate size analysis. Scale bars are 10 μm. **B**, Quantification of the diameter of GFP-Htt^ex1^-Q74 aggregates in (**A**). **C**, Detection of caspase 3/caspase 7 activity in pEGFP-Htt^ex1^-Q74 overexpressing PC-12 cells that were treated with IgG control or sHSPB5-Fc. The Caspases 3/7 activities were measured with the Caspase-Glo 3/7 kit. The bar graph represents data from three independent experiments. RLU, relative light units. Student’s t-test was used for the statistical analysis. *p < 0.05, **p < 0.01, ***p < 0.0001

According to the literature [39], reducing the size or increasing the solubility of Htt^ex1^-Q74 aggregates could reduce the cytotoxicity, Furthermore, we quantitated the diameter of GFP-Htt^ex1^-Q74 aggregates in the cells that were treated with normal IgG (sham) or sHSPB5-Fc using the Image J software. Approximately 150 GFP-Htt^ex1^-Q74 positive cells were used for analysis (Fig. 5A). The quantitative results showed that recombinant sHSPB5-Fc reduced the diameter of GFP-Htt^ex1^-Q74 aggregates compare to control IgG (Fig. 5B). These results suggest that sHSPB5-Fc protein can decrease the aggregate size of GFP-Htt^ex1^-Q74 in the PC-12 cell line.

The aggregation of GFP-Htt^ex1^-Q74 reportedly cause PC-12 cell cytotoxicity [40]. We have shown that sHSPB5-Fc can reduce the size and number of GFP-Htt^ex1^-Q74 aggregates. Therefore, we proposed that sHSPB5-Fc could reduce the cytotoxicity of GFP-Htt^ex1^-Q74 in PC-12 cells. To this end, PC-12 cells were transiently transfected with pEGFP- Htt^ex1^-Q74 vector and then treated with sHSPB5-Fc protein for 24 h. The activity of caspase 3 and caspase 7 were measured. The results showed that ectopic expression of GFP-Htt^ex1^-Q74 induced caspase 3/7 activation, and sHSPB5-F significantly decreased this activity (Fig. 5C). Taken together, these results suggest that sHSPB5-Fc protein can decrease the aggregation and cytotoxicity of Htt^ex1^-Q74 in PC-12 cells.

## Discussion

In this paper, we established a CHO-K1 subclone cell line that expresses and secretes human sHSPB5-Fc protein. This sHSPB5-Fc has both chaperone and anti-apoptosis activities, and can reduce the aggregation and cytotoxicity of mHtt proteins in vitro.

Delivery of HSPB5 proteins has shown a cytoprotective role against divergent stresses [41]. Most of the HSPB5 proteins used for in vivo administration are His-tagged HSPB5 generated via hetero-expression in the *E. coli* BL-21 strain [31, 32]. Although this is an economic way to obtain HSPB5, it is difficult to remove LPS contamination [42, 43], and the product cannot imitate some of the characteristics of mammalian HSPB5. To express sHSPB5 protein in mammalian cells, we utilized the CHO-K1-Fc cell system, a biomedical tool to express and produce recombinant proteins with an Fc-tag. We subcloned HSPB5 cDNA in-frame upstream of the Fc-tag. To improve the secretion of HSPB5-Fc protein, we added a secretion signal peptide sequence to the N-terminus of HSPB5. In this system, approximately 0.42 mg/ml of sHSPB5-Fc protein was obtained from the supernatant. We have shown that this recombinant sHSPB5-Fc protein is soluble, exists in dimer and trimer forms with glycosylation, and exhibits chaperone activity in vitro (Fig. 2). We further tested the cytoprotective role of this sHSPB5-Fc protein and found that it can inhibit cisplatin and UVB induced lens epithelial cell apoptosis. We also tested it neuroprotective roles in vitro, and found that administration of sHSPB5-Fc can reduce the size of the aggregates as well as the cytotoxicity of aggregated GFP-Htt^ex1^-Q74 in PC-12 cells (Figs. 3 and 4). These results suggest that sHSPB5-Fc protein possesses both chaperone and cytoprotective roles in vitro.

The Fc-tag, which is composed of hinge chain-CH2-CH3 domains of human IgG1 heavy chain, is a common tag used to express divergent recombinant proteins in the biomedicine industry [44, 45]. The FDA has approved 11 Fc-fusion proteins to treat different diseases, such as infectious diseases, cancer, and arthrosis [45]. In addition to facilitating protein-A-mediated immunoaffinity purification, Fc-tag also possess immune activity by binding to FcR, and activating the antibody-dependent cell-mediated cytotoxicity (ADCC) and complement systems [46]. A D143A mutation in the Fc-tag impairs its ADCC effect [47]. To get rid of the ADCC effect of sHSPB5-Fc, we mutated D143A in the Fc-tag portion of the sHSPB5-Fc protein. Mutation at this site does not affect HSPB5 chaperone (Fig. 1) or its cytoprotective activity in vitro (Fig. 2). Whether sHSPB5-Fc exhibits cytoprotective roles in vivo is still under investigation in our lab. Because of the Fc-tag, its potential regulation on the immune system should be considered when HSPB5-Fc is administrated in vivo.

The HSPB5 exists as a dimer or a polymer and its function depends on the dimer rather than polyoligomer [16]. To determine the status of sHSPB5-Fc, we treated sHSPB5-Fc in SDS loading buffer with or without β-ME, a reducer to destroy the disulfide bond. We found that the majority of sHSPB5-Fc protein is in dimer and trimer forms, and few sHSPB5-Fc forms polyoligomers (Fig. 1). β-ME destroyed the oligomers of sHSPB5-Fc to monomer (Fig. 1E), suggesting that disulfide bond may be involved in sHSPB5-Fc’ dimerization (Fig. 1E). But this needs more evidence (such as cysteine mutation in Fc) to confirm. Nevertheless, Fc does not impact HSPB5’s chaperone and cytoprotective activities (Figs. 2, 3, 4 and 5).

Glycosylation of proteins is an important posttranslational modification and can contribute to the folding and secretion of proteins [48]. In addition, glycosylation has complex significance on the function of proteins. For example, N-glycosylation can increase protein stability [35]. The N-glycosylation of Asn-297 of murine IgG can enhance immune response. Human recombinant lactoferrin, carrying humanized glycosylation, displays a selective antiproliferative effect on cancer cells [35]. Our data in Fig. 1 suggested that the HSPB5-Fc protein possesses an N-linked glycosylation, which may be beneficial to its biological activity. However, the effect of this glycosylation on HSPB5-Fc is unknown and needs further investigation.

## Conclusions

In summary, we established a mammalian expression system by using an industrialized CHO-K1 cell line, which can express and secret sHSPB5-Fc protein in a large scale. This sHSPB5-Fc protein is soluble in dimer or trimer. It can protect cells against cisplatin- or UV-induced cell apoptosis and attenuate the cytotoxicity of GFP-Htt^ex1^-Q74 aggregates in vitro.

## Methods

### Antibodies and chemical reagents

The rabbit antibody against HSPB5 was bought from Abcam (Cambridge, USA). The rabbit antibody against cleaved caspase 3 was from Cell Signaling Technology (Danvers, USA). The mouse antibodies against GFP and β-actin were from Proteintech (Wuhan, China). Phalloidin-iFluor 594 was from Abcam. Alexa Fluor 594 goat anti-rabbit IgG was from Thermo Fisher Scientific (Waltham, USA). Citrate synthase, DAPI and 302 serum-free medium were from Sigma (St. Louis, USA). PNGase F was from NEB (Beverly, USA). Caspase-Glo 3/7 assay kit was from Promega (Madison, USA). pEGFP-Htt^ex1^-Q74 plasmid was from Addgene (Cambridge, USA). HiTrap Protein A HP column was from GE Healthcare (Little Chalfont, UK).

### Cell culture

Chinese hamster ovary (CHO-K1) cells were maintained in our laboratory [47]. The cells were cultured in 302 serum-free medium supplemented with 100 U/mL penicillin, 100 mg/mL streptomycin (Gibco, Grand Island, USA) in an incubator with shaking. Mouse lens epithelial cells (mLEC) were cultured with DMEM medium (Gibco) supplemented with 10% fetal bovine serum (FBS, Gibco), 100 U/mL penicillin, and 100 mg/mL streptomycin. The PC-12 cell line was a gift from Xiao Xu, an Associate Professor at Henan University. The PC-12 cells were cultured in RPMI 1640 medium supplemented with 5% FBS, 10% horse serum (Gibco), 100 U/mL penicillin, and 100 mg/mL streptomycin.

### HSPB5-fc protein expression and purification

HSPB5 cDNA was synthesized by using RNA from human lens epithelial cells and subcloned into the pHB-Fc expression vector [47] at the HindIII (NEB, Beverly, USA) and EcoRI (NEB) restriction sites to generate pHB-HSPB5-Fc. To facilitate HSPB5-Fc secretion, A 57 bp cDNA fragment (ATGGGATGGTCATGTATCATCCTTTTTCTGGTAGCAACTGCAACTGGAGTACATTCA) encoding a signal peptide was inserted into the HindIII restriction site to fused to the 5′ terminal of HSPB5 cDNA (Supplementary sequence), generating the plasmid psHSPB5-Fc. The psHSBP5-Fc construct was transfected into and stably expressed in CHO-K1 cells. The secreted HSPB5-Fc (sHSPB5-Fc) recombinant protein in the supernatant was collected and purified with a HiTrap Protein A HP column in Akta pure 150 (GE Healthcare, Little Chalfont, UK).

For oligomerization of sHSPB5-Fc, the sHSPB5-Fc protein was heated in SDS-loading buffer (10% SDS, 0.5% bromophenol blue and 50% glycerol in 250 mM Tris-HCl, pH 6.8) with or without 5% β-Mercaptoethanol (β-ME) for 10 min. The samples were separated in SDS-PAGE gels followed by Coomassie blue staining. For glycosylation analysis, the sHSPB5-Fc protein was incubated with buffer containing PNGase F for 1 h at 37 °C. The proteins were separated in SDS-PAGE gel followed by Coomassie blue staining.

### Immunoblotting

Cells were lysed in RIPA buffer supplemented with protease inhibitor cocktail (Sigma) and phosphatase inhibitor cocktail (Sigma). Thirty to fifty microgram of proteins were separated by electrophoresis on SDS-PAGE gels, and then transferred to PVDF membranes. The membranes were blocked in 5% BSA/TBST for 1 h followed by incubating with the appropriate primary antibodies at 4 °C overnight. After washing three times in TBST buffer, the membranes were incubated in blocking buffer containing the appropriate HRP-conjugated secondary antibody. The membrane was washed in TBST buffer for 3–4 times and developed in ECL buffer. The signals were exposed to X-ray films.

### In vitro chaperone activity analysis

Citrate synthase (CS, 1 mg/ml) alone or combined with HSPB5-Fc was incubated at 43 °C in 40 mM HEPES buffer (pH 7.4) for the indicated times. The protein aggregates were measured at an absorbance wavelength of 320 nm in Varioskan Flash (Thermo electron corporation, Waltham, USA).

### Filter retardation assay

Cells expressing GFP alone or GFP-Htt^ex1^-polyQ74 were lysed with NP40 lysis buffer containing protease inhibitor cocktail. The cell lysates were centrifuged at 12,000 rpm/min for 5 min, and the supernatant was collected for western blot. The pellets were washed twice with NP40 lysis buffer followed by incubation with 10 U DNase I (NEB, Beverly, USA) in buffer containing 20 mM Tris-HCl, 15 mM MgCl_2_, pH 8.0 at 37 °C for 1 h to remove genomic DNA. After this, the pellets were combined with 2% SDS, 20 mM EDTA and 50 mM DTT and boiled at 98 °C for 5 min [49]. Fifty microgram of protein was filtered through a cellulose acetate (CA) membrane (0.2 μm) (Toyo Roshi Kaisha Ltd., Tokyo, Japan) using a slot blot unit (Midwestz, Beijing, China). The membranes were washed with 1% SDS and blocked in 5% non-fat milk/TBST. After this, the CA membrane was incubated with mouse anti-GFP antibody followed by HRP-conjugated goat anti-mouse secondary antibody. The membranes were developed in ECL buffer, and the signals were exposed to X-ray films.

### Immunofluorescent staining assay

PC-12 cells were transiently transfected with construct expressing GFP-Htt^ex1^-polyQ74 or pEGFP vector. The cells were fixed in 4% PFA for 20 min in room temperature followed by washing in PBS twice. The cells were then incubated with permeable buffer for 2 min and stained with buffer containing phalloidin-iFluor 594 (1:1000 dilution in PBS; Abcam) [50]. Nuclei were stained with DAPI (blue). The signals were visualized and captured using the confocal microscope (R1, Nikon, Japan).

### Annexin V/PI apoptosis assay by flow cytometry

Apoptosis was measured using an Annexin V-FITC/PI detection kit (KeyGEN BioTECH, Nanjing, China) following kit’s protocol. Briefly, cells were digested with non-EDTA trypsin. After washing with PBS, the cells were resuspended with binding buffer mixed with FITC-conjugated Annexin V and PI for 15 min at room temperature. After washing, the signal was measured by flow cytometry (BD Biosciences; San Jose, CA, USA) with excitation and emission wavelengths at 488 nm and 530 nm.

### Caspase 3/7 activity detection

The caspase 3/7 activity was measured by using the Caspase-Glo 3/7 kit (Promega, Madison, USA) following kit’s protocol. Briefly, the caspase 3/7 reagents were prepared by equally mixing the caspase 3/7 substrate and buffer freshly before use. One hundred microliter of mixed buffer was added to cells cultured in 96 well plates. After gently mixing, the plates were left RT for 1 h, and the luminescent signals, which reflect caspase-3/7 activities, were measured with Varioskan Flash (Thermo electron corporation, Waltham, USA).

### Statistical analysis

Image J was used to quantify the densitometry of immunoblot bands. SPSS 17.0 and GraphPad Prism 5 were used for data analysis. A Student’s t-test was used for statistical analysis. *p* < 0.05 was considered to be statistically significant.

## Supplementary Information

**Additional files 1: Figure S1.** The uncropped images of Figs. 1 and 2C. **Figure S2.** The uncropped images of Figs. 3 and 4C. **Figure S3.** The uncropped images of Fig. 4A. **Figure S4** The uncropped images of Fig. 5A. **Supplementary sequence.** The cDNA sequences for encoding recombinant HSPB5-Fc with signal peptide at N-terminal.

## Data Availability

All authors declare that the data supporting the findings of this study are available within the article and supplementary file.

## Abbreviations

sHSPB5-Fc: Secreted HSPB5-Fc
CS: Citrate synthase
CA: Cellulose acetate
ACD: Alpha-crystallin domain
β-ME: β-Mercaptoethanol
