# Supplementary material for: The engineered expression of secreted HSPB5-Fc in CHO cells exhibits cytoprotection in vitro
Source: BMC Biotechnol. 2021 Jun 14;21:39. doi: 10.1186/s12896-021-00700-y (PMC8204567; doi:10.1186/s12896-021-00700-y)
Supplement: Supplementary file 1 — Additional files 1: Figure S1. The uncropped images of Figs. 1 and 2C. Figure S2. The uncropped images of Figs. 3 and 4C. Figure S3. The uncropped images of Fig. 4A. Figure S4 The uncropped images of Fig. 5A. Supplementary sequence. The cDNA sequences for encoding recombinant HSPB5-Fc with signal peptide at N-terminal. [file 12896_2021_700_MOESM1_ESM.pdf]

The engineered expression of secreted HSPB5-Fc in CHO cells exhibits cytoprotection in vitro

Jing Li[1, 2, a], Jingjing Yu[1, a], Wenxian Xue[1, a], Huili Huang[1], Longjun Yan[1], Fan Sang[1], Shuangshuang An[1], Jing Zhang[1], Mingli Wang[1], Jun Zhang[1], Hui Li[1], Xiukun Cui[1], Jiang He[4] and Yanzhong Hu[1, 2, 3*]

[1] Joint National Laboratory for Antibody Drug Engineering, Henan International Union Lab of Antibody Medicine, Department of Cell Biology and Genetics, School of Basic Medical Sciences, Henan University, Kaifeng, China

[2] Kaifeng Key Lab for Cataract and Myopia, Institute of Eye Disease, Kaifeng Central Hospital, Kaifeng, China

[3] Department of ophthalmology, First Affiliated Hospital of Zhengzhou University, Zhengzhou, China

[4] Center for Molecular Medicine, Xiangya Hospital, Central South University, Changsha, China

a These authors contributed equally to this work.

* Corresponding author: Yanzhong Hu, Department of Cell Biology and Genetics, Henan University School of Basic Medical Sciences, Jin-Ming Road, Kaifeng, China, 475014; Email: hyz@henu.edu.cn; Tel. 86-18503781944; Fax.86-0371-23880398.

Figure S1 The uncropped images of figure 1 and 2C.

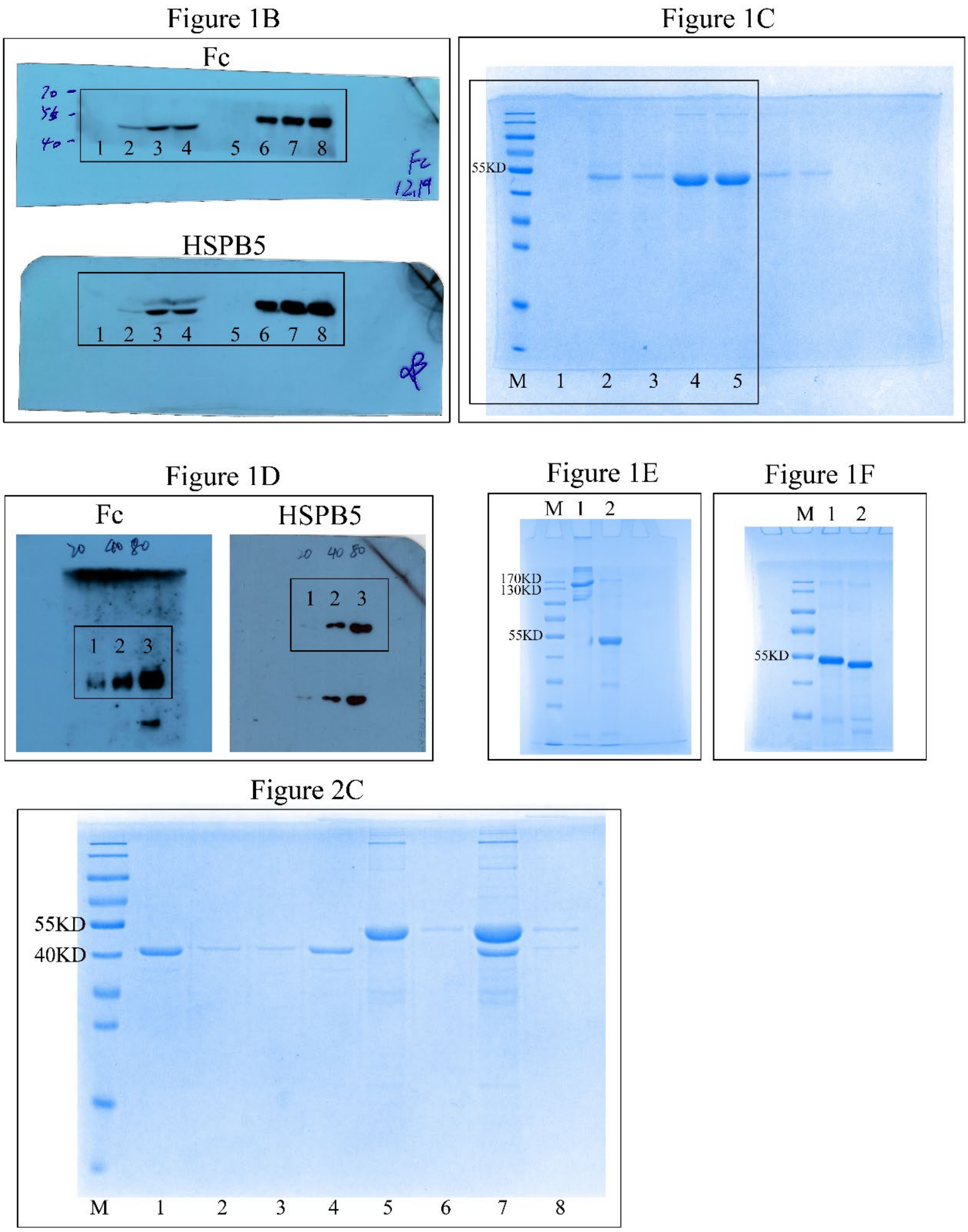

Figure S1 The uncropped images of figure 1 and 2C. Figure 1B, lanes 1-4 were supernatant of CHO-K1 cells. Lanes 5-8 were cell lysis of CHO-K1 cells. Lane 1: CHO-K1 cells with empty vector; lanes 2-5: CHO-K1 cells expressing three psHSPB5-Fc constructs (#1, #2, #3) respectively. Figure 1C, Coomassie blue stain of sHSPB5-Fc

protein purified from CHO-K1 supernatant (psHSPB5-Fc #2 construct). Lane 1 was the supernatant of CHO-K1 without vector. Lane 2 and 3 represented non purified sHSBP5-Fc in supernatants. Lane 4 and 5 represented purified sHSPB5-Fc. Figure 1D, Lanes 1-3 represents 20, 40 and 80ng sHSPB5-Fc proteins respectively. Figure 1E, sHSPB5-Fc protein was treated with SDS-loading buffer with (lane 2) or without (lane 1) β-mercaptoethanol. Figure 1F, sHSPB5-Fc protein was treated with (lane 2) or without (lane 1) PNGase F. Figure 2C, the soluble (lane 1) and pellet (lane 2) of citrate synthase (CS) protein at 4 °C. The soluble (lane 3) and pellet (lane 4) of citrate synthase (CS) protein at 43 °C. The soluble (lane 5) and pellet (lane 6) of sHSPB5-Fc protein at 43 °C. The soluble (lane 7) and pellet (lane 8) of CS with sHSPB5-Fc proteins at 43 °C. M: protein marker.

Figure S2 The uncropped images of figure 3 and 4C.

Figure 3A

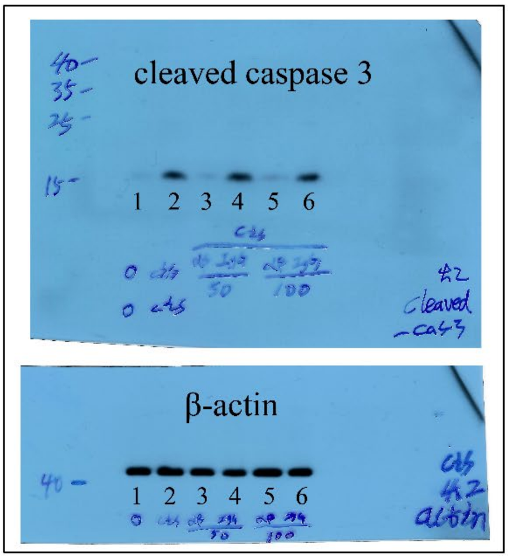

Figure 3D

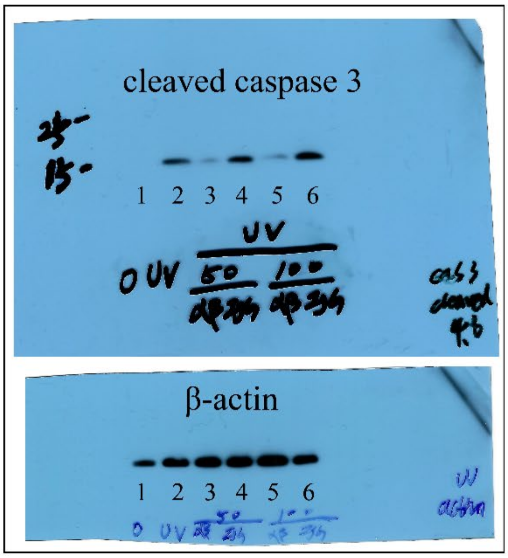

Figure 4C

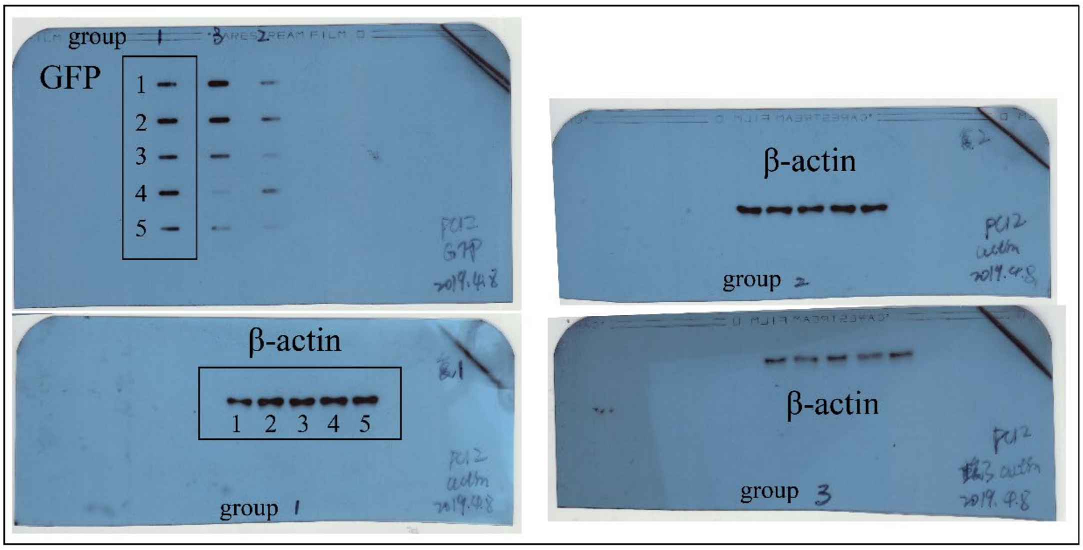

Figure S2 The uncropped images of figure 3 and 4C. Figure 3A, lane 1: mLEC cells without cisplatin treatment. Lane 2: mLEC cells with cisplatin alone. Lane 3: mLEC cells with cisplatin and 50 μg/ml sHSPB5-Fc. Lane 4: mLEC cells with cisplatin and 50 μg/ml human IgG. Lane 5: mLEC cells with cisplatin and 100 μg/ml sHSPB5-Fc. Lane 6: mLEC cells with cisplatin and 100 μg/ml human IgG. Figure 3D, lane 1: mLEC

cells without UV treatment. Lane 2: mLEC cells with UV alone. Lane 3: mLEC cells with UV and 50 μg/ml sHSPB5-Fc. Lane 4: mLEC cells with UV and 50 μg/ml human IgG. Lane 5: mLEC cells with UV and 100 μg/ml sHSPB5-Fc. Lane 6: mLEC cells with UV and 100 μg/ml human IgG. Figure 4C, the three results of membrane-filtration assays to detect the anti-aggregation properties of sHSPB5-Fc (groups 1-3). Lane 1: PC12 cells with pEGFP-Htt^{ex1}-Q74 vector. Lane 2: PC12 cells with pEGFP-Htt^{ex1}-Q74 vector and 50 μg/ml human IgG. Lane 3: PC12 cells with pEGFP-Htt^{ex1}-Q74 vector and 50 μg/ml sHSPB-Fc. Lane 4: PC12 cells with pEGFP-Htt^{ex1}-Q74 vector and 100 μg/ml human IgG. Lane 5: PC12 cells with pEGFP-Htt^{ex1}-Q74 vector and 100 μg/ml sHSPB-Fc.

Figure S3 The uncropped images of figure 4A

GFP DAPI

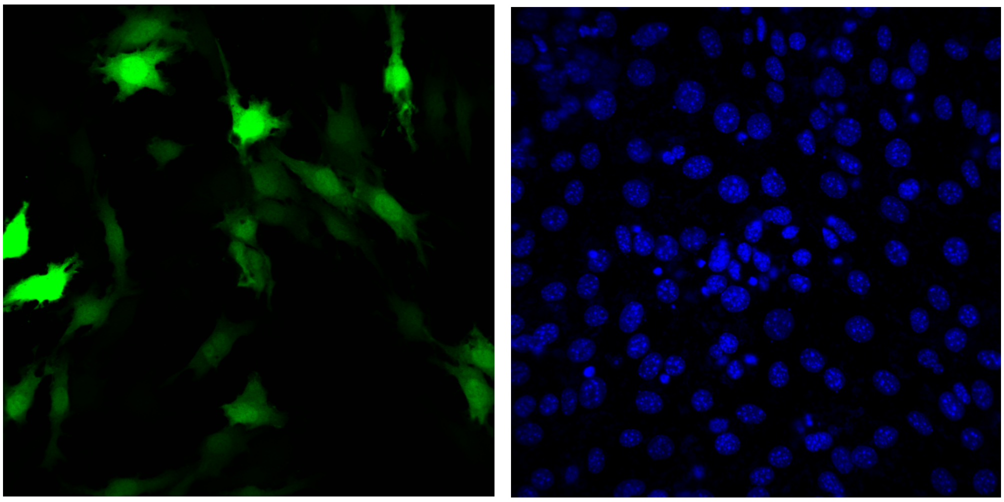

pEGFP vector

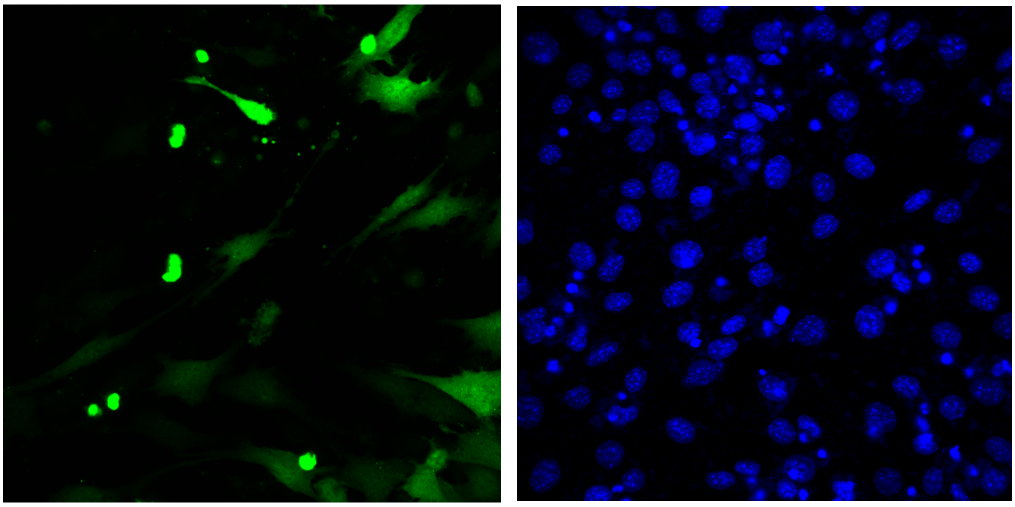

pEGFP-Htt^{ex1}-Q74+PBS

Figure S3 The uncropped images of figure 4A continued

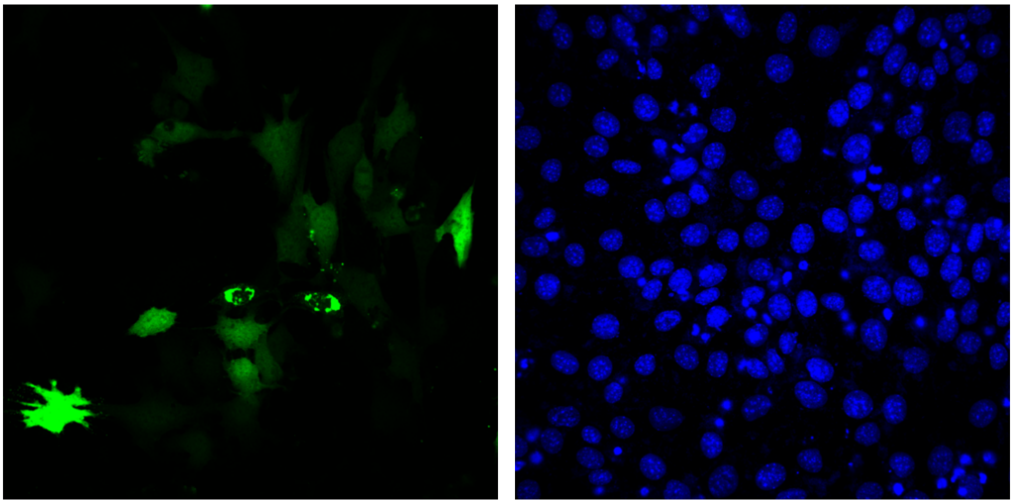

pEGFP-Htt^{ex1}-Q74+IgG 50 μg/ml

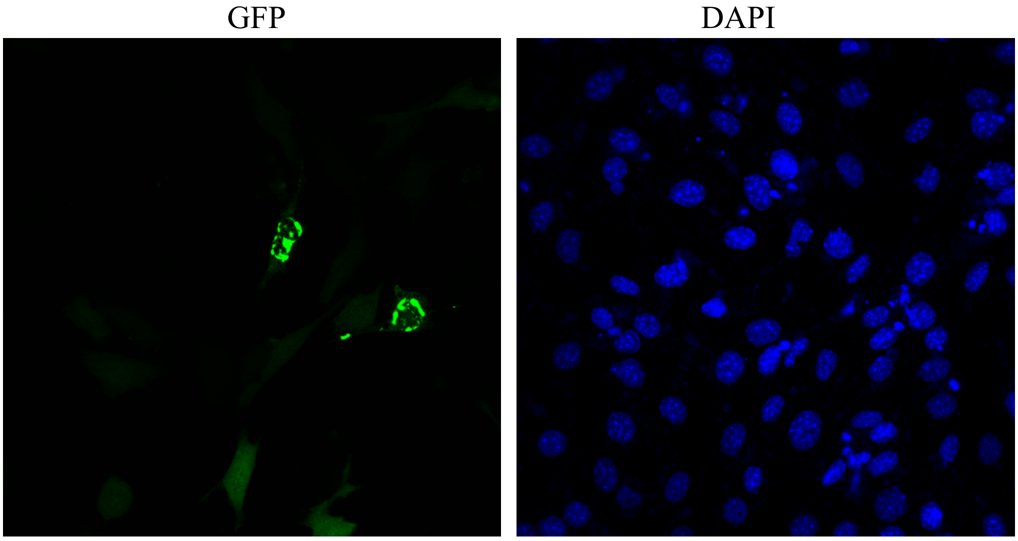

pEGFP-Htt^{ex1}-Q74+sHSPB5-Fc 50 μg/ml

Figure S3 The uncropped images of figure 4A continued

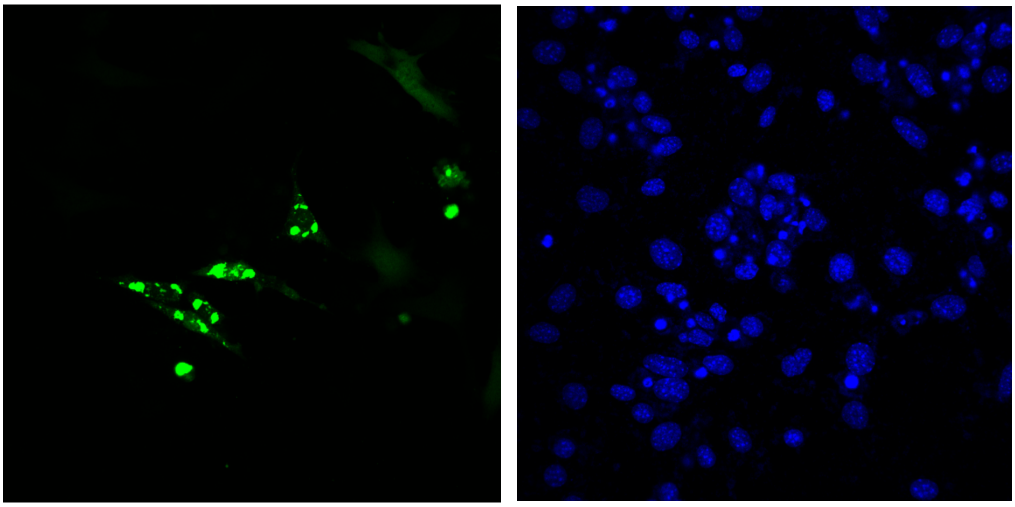

pEGFP-Htt^{ex1}-Q74+IgG 100 µg/ml

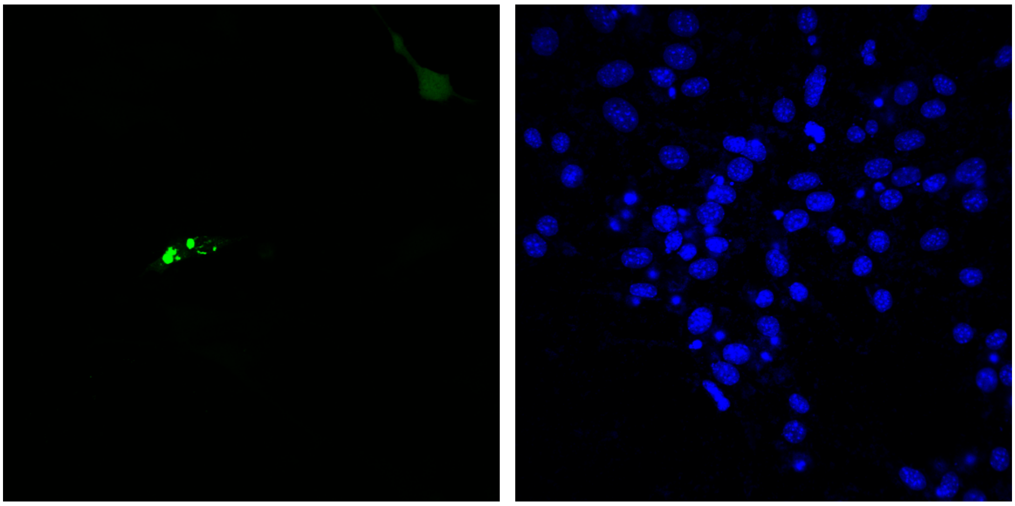

pEGFP-Htt^{ex1}-Q74+ sHSPB5-Fc 100 µg/ml

Figure S3 The uncropped images for figure 4A. Fluorescent image of PC-12 cells that express GFP alone or GFP-Htt^{ex1}-Q74. The cells were treated with human IgG or sHSPB5-Fc with 50 or 100 µg/ml.

Figure S4 The uncropped images of figure 5A.

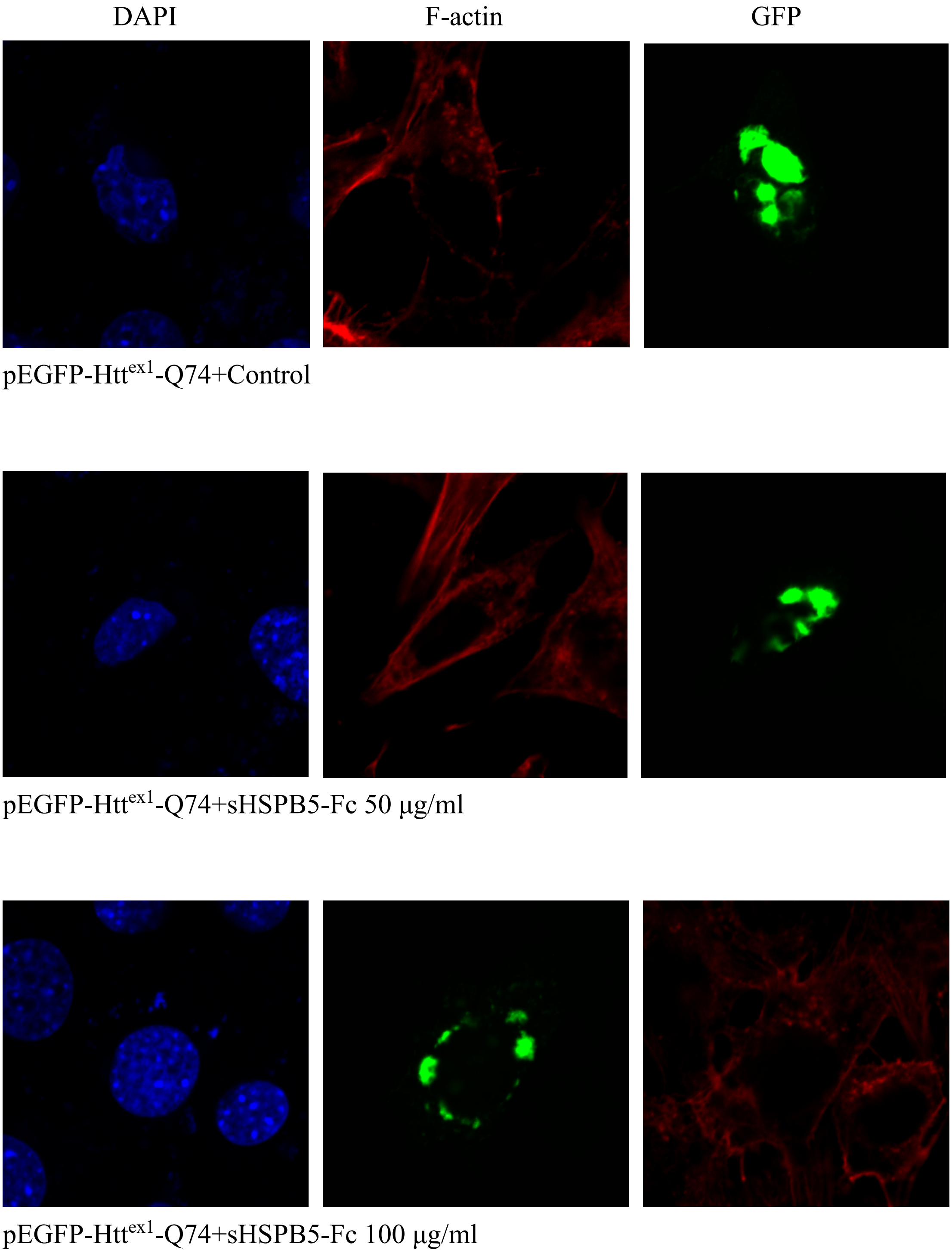

Figure S4 The uncropped images for figure 5A. PC-12 cells that express GFP-Htt^{ex1}-Q74 were treated with IgG control or sHSPB5-Fc.

Supplementary sequence

The cDNA sequences for encoding recombinant HSPB5-Fc with signal peptide at N-terminal

atgggatggtcatgtatcatcctttttctggtagcaactgcaactggagtacattcagacatcgccatccaccacccctggatc
caccgccccttctttcctttccactcccccagccgcctctttgaccagttcttcggagagcacctgttggagtctgatcttttccc
gacgtctacttccctgagtcccttctaccttcggccaccctccttcctgcgggcacccagctggtttgacactggactctcaga
gatgcgcctggagaaggacaggttctctgtcaacctggatgtgaagcacttctccccagaggaactcaaagttaaggtgttg
ggagatgtgattgaggtgcatggaaaacatgaagagcgccaggatgaacatggtttcatctccagggagttccacaggaa
ataccggatcccagctgatgtagaccctctcaccattacttcatccctgtcatctgatggggtcctcactgtgaatggaccaag
gaaacaggtctctggccctgagcgcaccattcccatcacccgtgaagagaagcctgctgtcaccgcagcccccaagaaa
gaattcgagcccaaatcttgtgacaaaactcacacatgcccaccgtgcccagcacctgaactcctggggggaccgtcagt
cttcctcttccccccaaaacccaaggacaccctcatgatctcccggacccctgaggtcacatgcgtggtggtggacgtgag
ccacgaagaccctgaggtcaagttcaactggtacgtggacggcgtggaggtgcataatgccaagacaaagccgcggga
ggagcagtacaacagcacgtaccgtgtggtcagcgtcctcaccgtcctgcaccaggactggctgaatggcaaggagtac
aagtgcaaggtctccaacaaagccctcccagcccccatcgagaaaaccatctccaaagccaaagggcagccccgagaa
ccacaggtgtacaccctgcccccatcccgggatgagctgaccaagaaccaggtcagcctgacctgcctggtcaaaggctt
ctatcccagcgacatcgccgtggagtgggagagcaatgggcagccggagaacaactacaagaccacgcctcccgtgct
ggactccgacggctccttcttcctctacagcaagctcaccgtggacaagagcaggtggcagcaggggaacgtcttctcatg
ctccgtgatgcatgaggctctgcacaaccactacacgcagaagagcctctccctgtctccgggtaaatga
